# Purine Catabolism Shows a Dampened Circadian Rhythmicity in a High-fat Diet-Induced Mouse Model of Obesity

**DOI:** 10.3390/molecules24244524

**Published:** 2019-12-10

**Authors:** Runbin Sun, Jingqiu Huang, Na Yang, Jun He, Xiaoyi Yu, Siqi Feng, Yuan Xie, Guangji Wang, Hui Ye, Jiye Aa

**Affiliations:** Jiangsu Provincial Key Laboratory of Drug Metabolism and Pharmacokinetics, State Key Laboratory of Natural Medicines, China Pharmaceutical University, Nanjing 210009, China

**Keywords:** metabolomics, circadian rhythms, high-fat diet, obesity, GC/MS, purine catabolism

## Abstract

High-calorie diet, circadian rhythms and metabolic features are intimately linked. However, the mediator(s) between nutritional status, circadian rhythms and metabolism remain largely unknown. This article aims to clarify the key metabolic pathways bridging nutritional status and circadian rhythms based on a combination of metabolomics and molecular biological techniques. A mouse model of high-fat diet-induced obesity was established and serum samples were collected in obese and normal mice at different zeitgeber times. Gas chromatography/mass spectrometry, multivariate/univariate data analyses and metabolic pathway analysis were used to reveal changes in metabolism. Metabolites involved in the metabolism of purines, carbohydrates, fatty acids and amino acids were markedly perturbed in accordance with circadian related variations, among which purine catabolism showed a typical oscillation. What’s more, the rhythmicity of purine catabolism dampened in the high-fat diet group. The expressions of clock genes and metabolic enzymes in the liver were measured. The mRNA expression of Xanthine oxidase (*Xor*) was highly correlated with the rhythmicity of *Clock*, *Rev-erbα* and *Bmal1*, as well as the metabolites involved in purine catabolism. These data showed that a high-fat diet altered the circadian rhythm of metabolic pathways, especially purine catabolism. It had an obvious circadian oscillation and a high-fat diet dampened its circadian rhythmicity. It was suggested that circadian rhythmicity of purine catabolism is related to circadian oscillations of expression of *Xor*, *Uox* and corresponding clock genes.

## 1. Introduction

Almost all living beings undergo circadian oscillations, which serve as a fundamental machinery to adapt physiological activities to environmental changes. This phenomenon, the circadian rhythm, is controlled by a circadian clock. Specifically, the suprachiasmatic nucleus (SCN) located in the hypothalamus is the most important circadian pacemaker in the central nervous system and is responsible for sensing light/darkness in a 24-h cycle to control the circadian rhythm [1,2]. The regulation of circadian rhythm is capable of influencing sleep, emotion, tumour development, metabolism, immunity, and reproductive function. Consequently, circadian disruption has been directly linked to diseases including obesity, diabetes, and cardiac disease [3,4,5,6,7,8]. A recent study demonstrated this relationship by showing that *Clock* mutant mice exhibited impaired diurnal feeding rhythms and developed metabolic syndromes, including hyperphagia, obesity, and hyperleptinemia [9]. Due to the pivotal role of the circadian clock in normal body functions, targeting circadian rhythms may be an effective therapeutic strategy for treating relevant diseases. In accordance with this notion, recent studies have reported that small molecule clock modulators can mediate circadian rhythmicity and improve metabolic functions including glucose and lipid metabolism, and thus benefit health [10,11,12]. While the influences of circadian rhythms on metabolism have been well recorded, it is intriguing to explore how metabolic processes in turn affect the circadian clock. Recent studies have shown that different nutritional statuses such as high-fat diets and ketogenic diets can affect circadian rhythms [13,14]. These discoveries all indicate a cause-effect relationship between altered energy homeostasis and circadian systems.

Obesity, one of the most prevalent diseases involving deregulated energy homeostasis, currently impacts nearly 37.7% of adults in the US [15]. In parallel with its continued increase, obesity has become a major health risk for public health. Previous investigations revealed a close relationship between obesity and circadian rhythms by showing that high-calorie diet-induced obesity leads to changes in the mammalian circadian clock by affecting the expression and oscillations of circadian clock genes and nuclear receptors that control clock transcription factors [16]. Consequently, it is intriguing to interrogate the interplay of obesity and circadian rhythms. The abnormal metabolic status of obesity might affect circadian clock genes, and subsequently alter circadian control of metabolic systems, which further disturbs normal metabolic physiological activities [9,16]. Metabolomics is by far the most sensitive technique to detect metabolic changes in a holistic view. It has recently been widely applied to characterize the abundance and dynamic changes in a myriad of metabolites in response to different physiological statuses and environmental stresses [17]. It can sensitively detect changes in metabolites involved in carbohydrate metabolism, fatty acid synthesis/degradation, amino acid biogenesis, and redox status [18,19]. Herein, we employed metabolomics to comprehensively describe the temporal oscillations of the metabolome from mouse sera and revealed the metabolic pathways that undergo the most pronounced changes in a group with high-fat diet-induced obesity compared with a normal group with a goal of identifying the hub that possibly sends a metabolic regulatory signal to the circadian system.

Therefore, we sought to describe the overall metabolomic changes in normal and high fat diet-induced obese mice during a 24-h cycle by gas chromatography coupled with mass spectroscopy (GC/MS) and to uncover the distinct rhythmicity of serum metabolome within a day. Furthermore, we measured the expression of canonical circadian clock genes, including *Rev-erbα,* Circadian Locomotor Output Cycles Kaput (*Clock*), brain muscle Arnt-like protein 1, Arntl (*Bmal1*), Cryptochrome 1/2 (*Cry 1/2*) and Period 1/2 (*Per 1/2*) in the liver. Among the complex clock gene network, *Clock* and *Bmal1* are positive modulation factors, while *Per1/2* and *Cry1/2* are negative modulation factors [20]. We also determined the expression of receptors or transcription factors that modulate metabolism. In conclusion, we sought to describe the relationship among nutritional status, metabolic oscillations, clock genes and specific metabolic enzymes.

## 2. Results

### 2.1. Feeding a High Fat Diet Induced Obesity in Mice

To investigate the influence of obesity on metabolic rhythmicity during a day, a HFD-induced obese mouse model was established. The body weights of the mice were recorded every week. After 8 weeks, the mice fed a HFD showed a significant increase in body weight (31.66 ± 2.04 g vs. 24.70 ± 1.55 g, *p* < 0.001) (Figure S1A). Meanwhile, the HFD did not affect the amount of food intake (Figure S1B). Interestingly, the serum glucose and cholesterol levels at each time point were all increased significantly compared with the normal diet group. Nevertheless, serum glucose showed a peak concentration at ZT8, while it gradually decreased during the light-dark cycle in mice fed both a normal diet and a HFD (Figure S1C). The serum cholesterol levels of the normal and HFD groups also showed similar upward trends during the daytime, followed by a modest decrease at night (Figure S1D).

### 2.2. Obesity Induced Significant Changes in the Circadian Serum Metabolome

**Figure 1:**
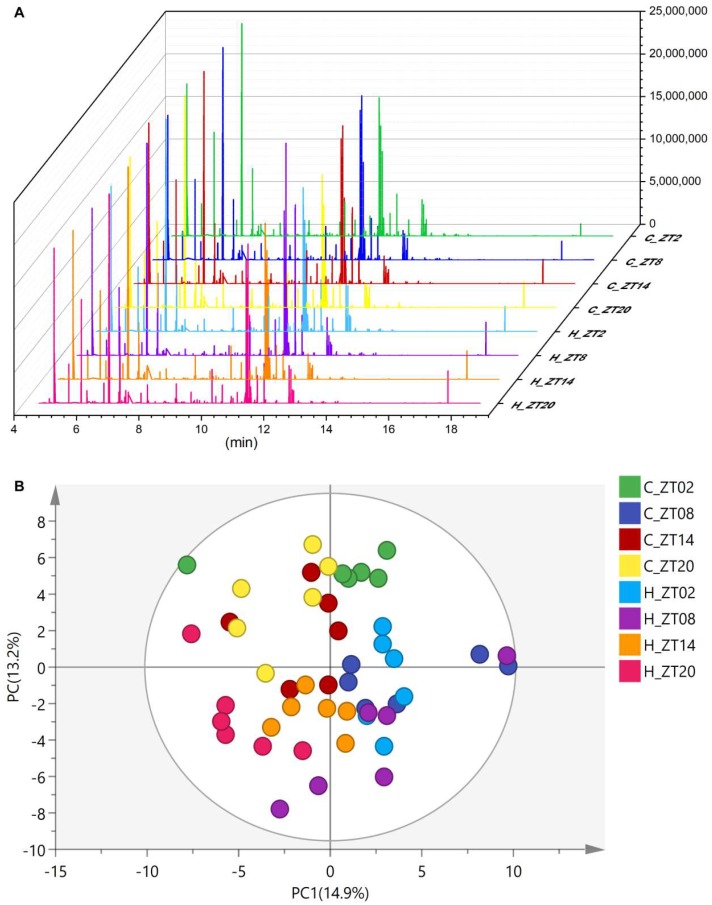
(**A**) Typical GC/MS chromatograms of serum from the normal diet group (C) and high fat diet group (H) at ZT2, ZT8, ZT14 and ZT20. (**B**) Scores plot of the principal component analysis (PCA) of mouse serum collected from the normal diet group (C) and the high fat diet group (H) at ZT2, ZT8, ZT14 and ZT20. Each point represents a metabolite profile of a biological replicate (*n* = 6).

Representative GC/MS chromatograms of the serum collected at each time point from both the normal diet group and the HFD group (4 sub-groups for each group, e.g., ZT2, ZT8, ZT14 and ZT20) are shown in Figure 1A. An unsupervised principal component analysis (PCA) model was applied to gain an overview of the circadian metabolome changes in response to HFD-induced obesity by analysing the metabolome of serum samples collected at different time points from the normal and HFD groups. No outlier was found using the PCA model (Figure 1B). A total of 105 peak features were integrated and 85 of them were identified, including amino acids, organic acids, carbohydrates, fatty acids, purines, etc.

**Figure 2:**
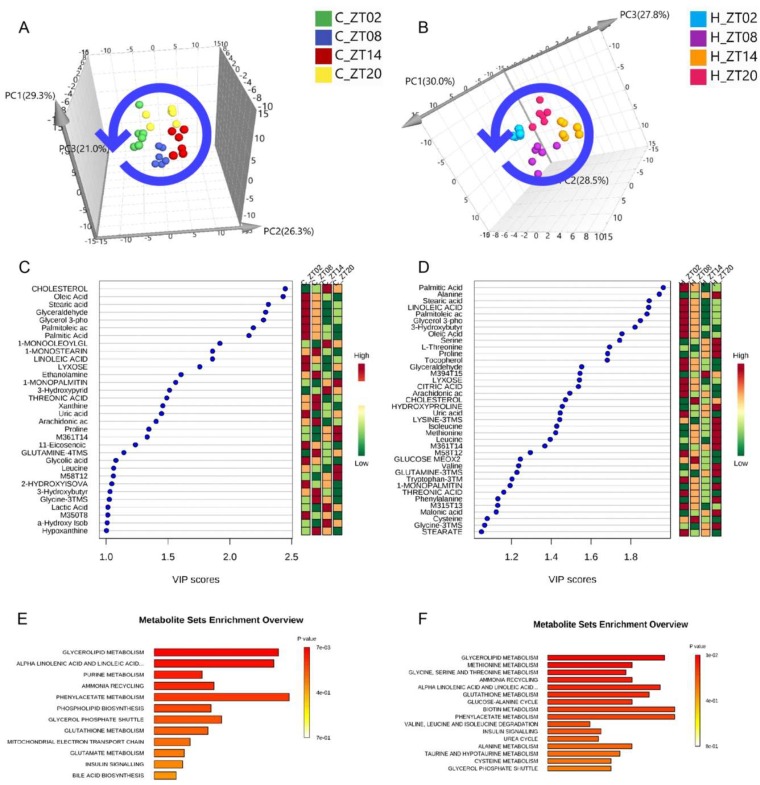
Metabolic impact analysis based on GC/MS analysis of the serum metabolome the of normal diet group (C) and the high-fat diet group (H) mice at ZT2, ZT8, ZT14 and ZT20. (**A**) Score plots of the partial least squares discriminant analysis (PLS-DA) of serum samples from normal mice collected at different time points during a day. (**B**) Score plots of PLS-DA of serum samples from mice given high-fat diet collected at different time points during a day. (**C**) Variable importance in the projection (VIP) analysis of serum samples from normal mice collected at different time points during a day. The relative concentrations of the compounds in each sub-group are represented by green, yellow and red, from low to high. (**D**) VIP analysis of PLS-DA of serum samples from mice given a high-fat diet collected at different time points during a day. (**E**) Metabolite pathway enrichment analysis based on metabolites that displayed significant circadian oscillations from the normal group. (**F**) Metabolite pathway enrichment based on metabolites that displayed significant circadian oscillations from the mice given a high-fat diet.

A partial least squares discriminant analysis (PLS-DA) model was then employed for the identification of potential metabolic biomarkers that differentiate the two groups. Consequently, we first investigated the levels of metabolic changes in samples from the normal diet group and the HFD-fed groups collected at different time points, and then interrogated whether obesity further influenced the rhythmicity of the observed circadian metabolome. As shown in Figure 2A, the 3D scores plot showed that the serum samples obtained at the same time point were clustered together and segregated from those at different time points, indicating metabolic oscillations over time. Furthermore, a time-dependent shift in the serum metabolome was observed from ZT2 to ZT20. A similar trend was also seen for the sub-groups in the HFD group (Figure 2B). This suggested that circadian rhythms affect the serum metabolome of both normal and obese mice, and that the HFD further influenced the circadian rhythmicity of the normal metabolome.

### 2.3. Metabolic Biomarkers Affected by Circadian Rhythms in Normal Mice and High-Fat Diet-Induced Obese Mice

To determine the identities of serum metabolites associated with circadian clock, the metabolic profiles of mouse serum samples were analysed by PLS-DA and VIP analysis revealed 32 compounds (five compounds not identified) in the normal diet group and 38 compounds (four compounds not identified) in the HFD group with a VIP score >1 (Figure 2C,D). These compounds were further classified as metabolites involved in glycerolipid metabolism; α-linoleic acid and linoleic acid metabolism; purine metabolism; ammonia recycling; phenylacetate metabolism; phospholipid biosynthesis; etc. in the normal diet group (Figure 2E,F) and as metabolites involved in glycerolipid metabolism; methionine metabolism; glycine, serine and threonine metabolism; ammonia recycling; alpha-linoleic acid and linoleic acid metabolism; glutathione metabolism; etc. in the HFD group by metabolic pathway enrichment analysis. Interestingly, we also found 18 metabolic biomarkers of circadian oscillations that overlapped between the normal diet and HFD groups, which were mostly fatty acids and amino acids. A total of nine metabolites, including lactic acid, 1-monostearin and 2-hydroxyisovaleric acid, only oscillated in the normal diet group, whereas 16 metabolites including citric acid and amino acids were only altered in the HFD group (Figure S2).

**Figure 3:**
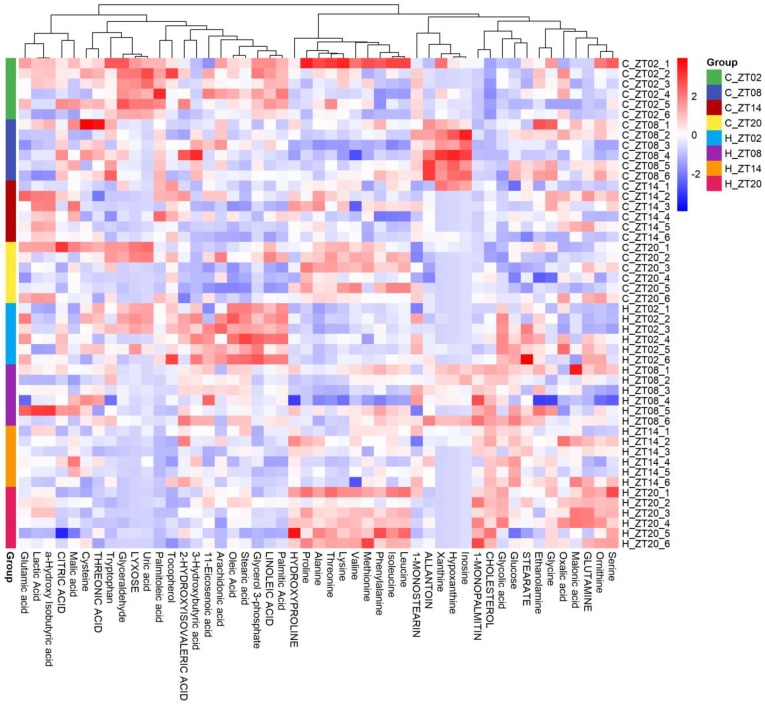
Heatmap showing the relative amount of individual metabolites. The order of the metabolite was determined by hierarchical clustering. Blue colour represents a lower while red colour represents a higher amount.

Furthermore, a heatmap of all the differentially expressed individual metabolites was created (Figure 3). Metabolites that had similar trends could be clustered into one category by unsupervised hierarchical clustering. Intriguingly, metabolites from the same biochemical pathway were clustered together. For example, amino acids were clustered in one matrix, while fatty acids were clustered in another matrix and showed opposite trend with amino acids. The intermediates of the Krebs cycle, such as citric acid and malic acid, were clustered together and had opposite trend with amino acids while similar with fatty acids. Such trends can be explained by the interconversions between fatty acids and gluconeogenic substrates through oxidation/reduction and transamination. Moreover, the intermediates of purine catabolism, inosine, hypoxanthine, xanthine and allantoin were clustered together and highly correlated with each other (Figure 3).

### 2.4. Remodelling Metabolic Circadian Rhythms by High-Fat Diet-Induced Obesity

**Figure 4:**
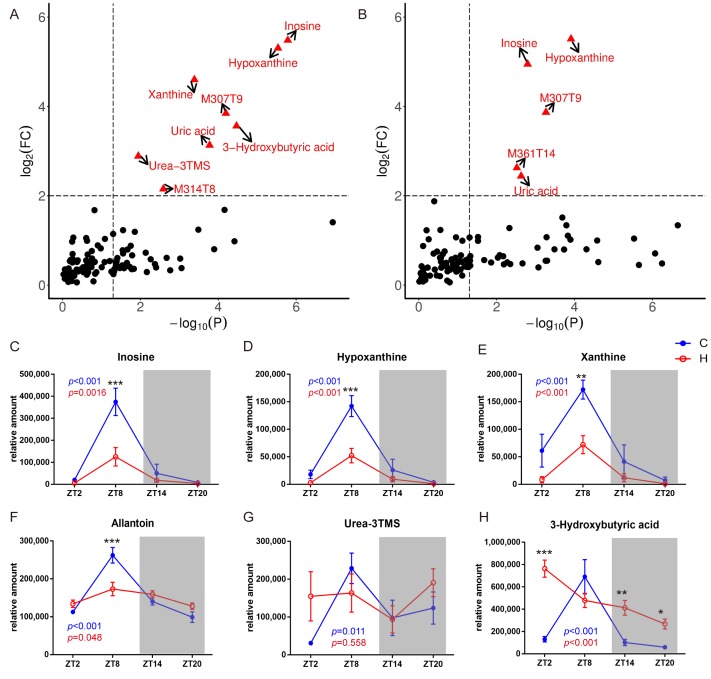
Fold change (FC) of the metabolites between highest and lowest concentration at different time points in the serum of mice from the normal diet group (**A**) and the high-fat diet group (**B**). Line graphs show the normalized abundance of metabolites at ZT2, ZT8, ZT14 and ZT20, respectively. Data are represented as the mean ± SD. (**C**–**H**) *p* value in blue colour, one-way ANOVA of four groups under normal diet; *p* value in red colour, one-way ANOVA of four groups under HFD diet.

Based on metabolic pathway enrichment analysis and differential metabolite analysis, we summarized the metabolites that undergo circadian oscillations (Figure 2 and Figure 3). Furthermore, univariate data analysis was conducted to find the most oscillated compounds (Figure 4A,B). Notably, metabolites involved in purine catabolism, including inosine, hypoxanthine, xanthine and allantoin (Figure 4C–F) all showed impaired rhythmicity by the decreasing peak concentration present at ZT8 in the HFD group. Furthermore, curves of metabolites were fitted using trigonometric function, and the amplitudes of the HFD group were all decreased compared with the normal diet group (Figure S3). This is in accordance with the previous study [21]. Meanwhile, serum uric acid also lost its rhythmicity in the obese group compared with the normal diet group. It peaked at ZT2, decreased during the daytime and increased back to the peak concentration at night. Serum urea peaked at ZT8 in the normal diet group, and lost its rhythmicity in the obese group (Figure 4G).

Interestingly, the diurnal rhythmicity pattern of a ketone body, 3-hydroxybutyric acid, was completely changed in the HFD-induced obese mice. Specifically, this metabolite peaked at ZT8 in the serum of the normal diet group. However, the level of 3-hydroxybutyric acid displayed a prominent cycle with the lowest concentration observed at ZT20 in the HFD group. In addition, 3-hydroxybutyric acid showed a generally higher level in the HFD group than in the normal diet group at all the time points except ZT8 (Figure 4H).

Marked differences between the cycling of carbohydrate metabolism and the Krebs cycle in the normal and HFD-induced obese groups were observed. Specifically, glucose showed a peak concentration at ZT8 and decreased at ZT20 in the serum of the normal diet group, whereas the concentration of glucose in the serum of the HFD group was increased at all the time points compared with the normal diet group and partially lost rhythmicity by decreasing the differences between the highest and lowest concentrations of glucose within a day. This observation was consistent with the results measured by the glucose kit (Figure S1C) and previous literature [22]. The concentration of pyruvic acid oscillated during a day by reaching its highest and lowest levels at ZT8 and ZT14 for the normal diet group and the HFD group, respectively. The concentration of lactic acid also oscillated during a day by reaching its highest and lowest levels at ZT8 and ZT14, respectively. The HFD weakened this rhythmicity and changed the circadian profile by reaching a rather stable plateau from ZT8 to ZT20. In contrast, the level of citric acid was rarely influenced by the circadian clock in the serum of the normal diet group while it showed more oscillatory changes in the HFD group by exhibiting its lowest concentration at ZT20. (Figure S4A)

In addition to carbohydrate metabolism and the Krebs cycle, lipid metabolism also showed changes throughout the day. For fatty acids, oleic acid, palmitic acid, palmitoleic acid, stearic acid, linoleic acid and arachidonic acid all showed a similar diurnal rhythmicity, i.e., exhibiting a cyclic oscillation between ZT2 and ZT20, in both groups. Nevertheless, oleic acid and stearic acid were both significantly increased by HFD in mouse serum collected at each time point, whereas the concentration of palmitoleic acid was decreased in the HFD group compared with the normal diet group (Figure S4B). Glycerol-3-phosphate is an intermediate that participates in both carbohydrate and lipid metabolism. It had a peak concentration at ZT2, gradually reduced during the daytime and increased again at night. Interestingly, the HFD had no influence on glycerol-3-phosphate as shown in Figure S4B.

Regarding amino acids, alanine, methionine, leucine, isoleucine and valine all peaked at ZT20 in both groups, while HFD merely slightly increased the rhythmicity of these amino acids. In the normal diet group, serum glutamine tended to peak at ZT14, whereas the HFD treatment significantly broke the rhythmicity by upregulating the level of glutamine at night and reaching the peak at ZT20. Tryptophan, serine and threonine showed similar circadian oscillation profiles by displaying peaks at day time in both the normal diet group and the HFD group (Figure S4C).

### 2.5. Circadian-Related Expression of Xanthine Oxidase and Uricase

**Figure 5:**
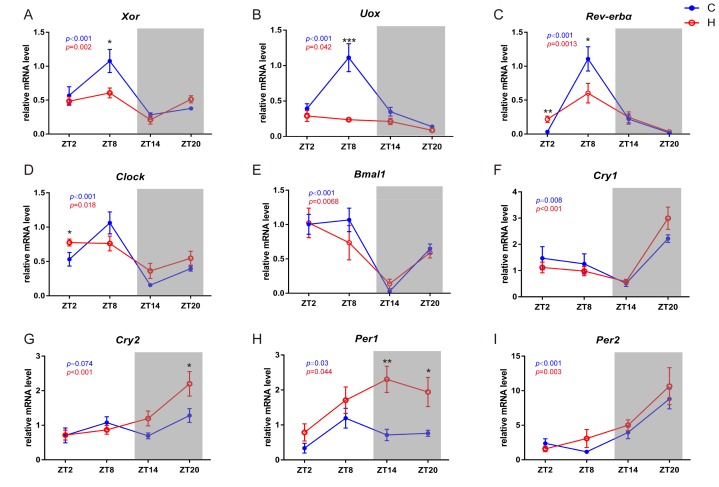
Circadian expression of metabolic enzymes, *Xor* (**A**), *Uox* (**B**) and clock genes, including *Rev-erbα* (**C**), *Clock* (**D**), *Bmal*1 (**E**), *Cry1* (**F**), *Cry2*(**G**), *Per1* (**H**) and *Per2*(**I**), in the liver of mice from the normal diet group (**C**) and the high-fat diet group (**H**) at ZT2, ZT8, ZT14 and ZT20. Line graph shows the relative mRNA levels of target genes. Data are represented as the mean ± SD. *, *p* < 0.05 **, *p* < 0.01 ***, *p* < 0.001, compared with the normal diet group at each time point. *P* value in blue colour, one-way ANOVA of four groups under normal diet; *p* value in red colour, one-way ANOVA of four groups under HFD diet.

Since the rhythmicity of metabolites involved in purine catabolism was significantly decreased in the HFD group, we thus asked whether such changes in the serum metabolome are reflected by the changes in metabolic enzymes that are responsible for the corresponding metabolic reactions. Xanthine oxidase (*Xor*), which catalyses the oxidation of hypoxanthine to form xanthine and of xanthine to form uric acid and Urate Oxidase (*Uricase*, *Uox*), which is an enzyme that converts uric acid into allantoin, were selected as representative enzymes. Since all metabolites detected involved in catalysis by *Xor* and *Uox* showed impaired rhythmicity, we performed qRT-PCR analysis of liver samples and found that the mRNA levels of *Xor* (Figure 5A) and *Uox* (Figure 5B) were also regulated by circadian rhythms. Furthermore, curves of metabolic enzymes were fitted using trigonometric function, and the amplitudes of the HFD group were all decreased compared with the normal diet group (Figure S5A,B). The oscillatory patterns of *Xor* and *Uox* were consistent with the corresponding metabolic changes, thereby explaining the decreased rhythmicity of hypoxanthine, xanthine, uric acid and allantoin.

**Figure 6:**
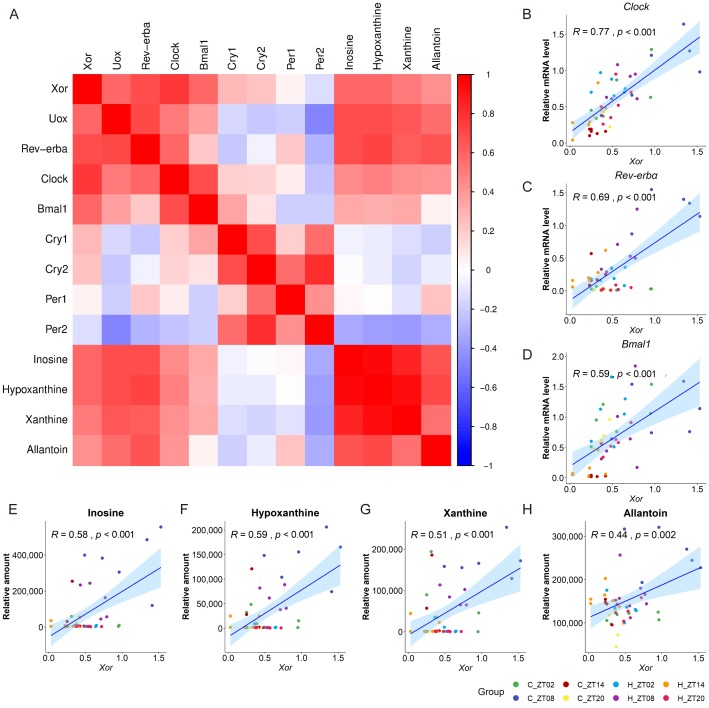
The heatmap (**A**) showing the correlation coefficients between *Xor* and individual clock genes. Each square represents the Pearson’s correlation coefficient between the metabolite of the row and that of the column. Red colour represents a positive correlation, while blue coluor represents a negative correlation with other metabolites. The correlation coefficients between *Xor* and individual clock genes, *Clock* (**B**), *Rev-erbα* (**C**), *Bmal*1 (**D**), and the correlation coefficients between *Xor* and metabolites involved in purine catabolism, inosine (**E**), hypoxanthine (**F**), xanthine (**G**) and allantoin (**H**).

Since previous studies suggest that a myriad of metabolic enzymes are under circadian control by clock genes [20,23,24], we also measured several canonical clock genes in the liver by qRT-PCR including *Rev-erbα* (Figure 5C), *Clock* (Figure 5D), *Bmal1* (Figure 5E), *Cry1/2* (Figure 5F,G), and *Per1/2* (Figure 5H,I). Canonical correlation analysis showed that the canonical correlation coefficients of the first three couples of canonical variables representing the concentration of metabolites involved in purine catabolism and the expression of circadian genes were 1.00, 0.91 and 0.80, respectively, and the *p* values were all less than 0.05. Furthermore, curves of clock genes were fitted using trigonometric function, and the amplitudes of the HFD group were all decreased compared with the normal diet group (Figure S5C–F). These results indicated that the concentrations of metabolites involved in purine catabolism were closely correlated with the expression of circadian genes. To reveal the relationship between *Xor* and the clock genes, correlation analysis between them was conducted. The heatmap (Figure 6A) showed the correlation coefficients between *Xor* and individual clock genes. Interestingly, we found the oscillatory expressions of *Xor* was positively related to the expression of *Clock* (Figure 6B), *Rev-erbα* (Figure 6C) and *Bmal1* (Figure 6D) and the concentrations of inosine (Figure 6E), hypoxanthine (Figure 6F), xanthine (Figure 6G) and allantoin (Figure 6H). These data suggested that the regulation of the expression and activity of *Xor* might be clock-related. In accordance with the reduced circadian rhythms induced by HFD deduced from the metabolomics results, we observed a similar trend of reduced rhythmicity in the expression levels of clock genes, except for *Per1*.

## 3. Discussion

Metabolomics has become an emerging technique to comprehensively reveal changes in endogenous metabolites with great sensitivity and high throughput. It is widely applied to disease prognosis and diagnosis, drug efficacy evaluation, biomarker discovery, etc. [25,26,27,28,29,30]. Previously, it has been reported that nearly all fundamental physiological activities show circadian rhythms. A previous investigation employed GC/LC coupled with MS and indicated that, in human plasma, 15% of total metabolites were under circadian control [18]. In addition, a molecular-timetable method has been established and used to quantify clock-controlled metabolites in mouse plasma to measure internal body time [31]. Consequently, we employed metabolomics to profile metabolite cycling and specifically focused on obesity-induced changes in the circadian oscillations of the serum metabolome within a 24-h cycle.

Our results also revealed that endogenous metabolism was closely associated with circadian rhythm. Moreover, some serum metabolites exhibited almost identical cycling patterns in the normal diet and HFD groups. For example, glycerol-3-phosphate showed peak concentrations at ZT2 in both the normal diet group and the HFD group. Its rhythmicity can be explained by previous findings that reported multiple enzymes involved in the glycerol-3-phosphate pathway--the expression of glycerol-3-phosphate acyltransferase 2 (GPAT2) peaked at circadian time (CT, endogenous rhythm) 0 and decreased at CT12, while 1-acylglycerol-3-phosphate acyltransferase 1/2 (AGPAT1/2) transcripts also accumulated in a circadian manner, with Agpat1 peaking at CT16 and Agpat2 peaking at CT0 [32]. In addition, some metabolites, such as glucose, exhibited similar oscillation profiles and decreased rhythmicity in response to a HFD. The different circadian oscillatory profiles of glucose indicate the deregulated homeostasis of glucose, while the loss of rhythmicity can be attributed to enhanced glucose production by the liver and decreased consumption by muscle, fat, brain, etc. Lipid metabolism is also tightly associated with circadian rhythm. The identified serum fatty acids all showed peak concentrations in the morning and were greatly reduced at other times of the day. Regarding the HFD-induced changes, although the HFD increased the concentrations of most identified fatty acids, it resulted in no marked changes in their circadian oscillatory profiles. However, whether these effects were circadian controlled or merely the influence of the diet need to be further investigated.

As for amino acids, the circadian rhythms of branched chain amino acids (BCAA), including valine, isoleucine and leucine, were slightly affected by the HFD treatment. All three branched chain amino acids reached their highest concentration at ZT20, which is consistent with the results of previous studies [33,34]. BCAAs have previously been suggested to be predictive of diabetes and insulin resistance based on their plasma levels [35,36], while our study suggests that their changed rhythmicity might be a new marker of interrupted metabolic homeostasis in addition to static expression levels. We also found that, in the serum of the control group, tryptophan decreased at ZT14. Nevertheless, this rhythmicity was weakened by HFD. Tryptophan has the ability to affect the sleep/wake cycle by mediating the metabolism of the neurotransmitter serotonin, which can be converted to melatonin in the pineal gland and thus regulate sleep. Consequently, a disrupted sleep cycle is noted in patients with obesity [16,37]. Glutamine is the most abundant amino acid present in plasma and can be transported across the blood-brain-barrier followed by converted into glutamate, a powerful excitatory neurotransmitter [38]. In sleep-deprived rodents, glutamine was increased in the posterior hypothalamic region. Glutamine is also associated with reactive-oxygen species (ROS) production [39]. In this study, we noticed that HFD increased the rhythmicity of glutamine, i.e., glutamine reached its peak concentration at ZT20, which suggests that the mice fed a HFD are prone to receive more excitatory signals at night than mice fed with a normal diet.

In humans, purines are metabolized to hypoxanthine and xanthine and finally to uric acid, whereas in rodents uric acid was further metabolized to allantoin by *Uox*. *Xor* is the rate-limiting enzyme responsible for the oxidation of hypoxanthine and xanthine [40]. Abnormal levels of uric acid have been associated with gout, a kind of inflammatory arthritis [41], and disturbed purine catabolism was observed in patients with metabolic syndrome, cardiovascular disease and type 2 diabetes [42,43,44,45]. Previous studies have also revealed that the level of uric acid is circadian-related [46]. Additionally, the upstream metabolite of uric acid, adenosine, can function as a molecular oscillator to regulate circadian rhythms [47]. Our observation of the decreased rhythmicity of metabolites involved in purine catabolism thus suggests that HFD-induced obesity interrupted circadian rhythms and deregulated the circadian clock. This was further confirmed by the altered circadian oscillations of canonical clock genes, such as *Clock*, *Bmal1* and *Rev-erbα*, as shown in Figure 5. Another interesting discovery is the positive correlation of *Xor* expression with clock genes, indicating a potential functional interplay between clock gene machinery and purine catabolism. In accordance with our findings, previous research reported the regulation of *Xor* by circadian control in the marine alga *Gonyaulax* [48], while this phenomenon has not been demonstrated in mammals. However, the direct regulation of Xor by clock genes needs to be further investigated. Furthermore, previous studies revealed that the timed administration of drugs without changing dosage can improve the potency of anti-inflammatory drugs and anti-tumor drugs [49]. We speculated that the effect of allopurinol on treating gout may be affected by the dosing time, which should be further validated.

## 4. Materials and Methods

### 4.1. Chemicals

Stable-isotope-labelled [^13^C_2_]-myristic acid was purchased from Cambridge Isotope Laboratories (Andover, MA, USA). Methoxyamine and a mixture of *N*-methyl-*N*-trimethylsilyl-trifluoro-acetamide (MSFTA) and 1% trimethylchlorosilane (TMCS), avertin (2,2,2-tribromoethanol) and 2-methyl-2-butanol were purchased from Sigma-Aldrich (St. Louis, MO, USA). Methanol and *n*-heptane (chromatography grade) were purchased from Merck KGaA (Darmstadt, Germany).

### 4.2. Animal Studies

Forty-eight male C57BL/6J mice (six weeks old, purchased from Yangzhou University, Yangzhou, China) were housed under 12-h light/12-h dark conditions (lights on at 6:00 a.m. and off at 6:00 p.m., ZT0 and ZT12, respectively) at constant room temperature (25 ± 2 °C). All mice were fed a standard chow diet (AIN-93M, Trophic Animal Feed High-Tech Co., Ltd., Nantong, China) and tap water *ad libitum* for one week to acclimate them to the environment. The mice were divided into two groups (*n* = 24), the normal group (C) and the high-fat diet group (H). The normal group was fed a standard chow diet continuously for 8 weeks, and the high-fat diet group was fed with high-fat diet (HFD, 60% calories from fat and containing 1% cholesterol, Trophic Animal Feed High-Tech Co., Ltd., Nantong, China) for 8 weeks. The nutritional compositions of AIN-93M and HFD are listed in Table 1.

By the end of the experiment, each group of mice was randomly divided into 4 sub-groups (*n* = 6) and sacrificed at zeitgeber time (ZT) 2 (8:00), ZT8 (14:00), ZT14 (20:00), and ZT20 (2:00). At the time of sacrifice, the mice were first anaesthetized by avertin (2.5 g of 2,2,2-tribromoethanol, 5 mL of 2-methyl-2-butanol, and 200 mL of distilled water) at a dose of 10 mL/kg, while the serum from each animal was prepared by centrifuging the collected whole blood at 8,000 rpm for 10 min, and the livers were harvested and stored at −80 °C for further analysis.

### 4.3. Measurement of Serum Glucose and Cholesterol

The levels of serum glucose and cholesterol were measured using commercial kits according to the manufacturers’ instructions (purchased from Nanjing Jiancheng Bioengineering Institute, Nanjing, China).

### 4.4. Sample Preparation and GC/MS Analysis

A GC/MS-based metabolomics method was used to profile metabolites from the serum of each group as previously reported [50]. Briefly, 50 μL of serum was extracted with 200 μL of methanol containing 5 μg/mL of [^13^C_2_]-myristic acid as an internal standard (IS) and centrifuged at 20,000× *g* for 10 min. An aliquot of 100 μL of supernatant was collected and concentrated to dryness using a SpeedVac (Thermo Fisher Scientific, Pittsburgh, PA, USA). The extract was then oximated with 30 μL of pyridine containing 10 mg/mL methoxyamine for 16 h at room temperature, followed by derivatization with 30 μL of MSTFA + 1% TMCS for 1 h. Subsequently, 30 μL of *n*-heptane was added and thoroughly mixed. A QP2010 Ultra/SE GC/MS system (Shimadzu, Kyoto, Japan) with an RTx-5MS fused silica capillary column (30 m × 0.25 mm ID, J&W Scientific, Bellefonte, PA, USA) was used for metabolomics analysis.

### 4.5. Compound Identification and Statistical Analysis

The approach of compound identification was the same as previously described [25,51]. The metabolites were identified using the National Institute of Standards and Technology (NIST) library 14, Wiley 9 and an in-house mass spectra library database. The raw data acquired by GC/MS were processed by GCMS solution ver. 4.11 (Shimadzu), and the peak areas of detected compounds were normalized to that of the IS for each sample. PCA and PLS-DA were performed using SIMCA-P 13.0 software (Umetrics, Umeå, Sweden). The PLS-DA model was used to confirm the general separation among the four groups, and variable importance in the projection (VIP) analysis was used to identify the endogenous metabolites contributing to the classification. The strength of a pattern recognition model was evaluated by the parameters R^2^X or R^2^Y and Q^2^Y parameters. Metabolomics pathway analysis of the metabolic biomarkers was carried out using MetaboAnalyst (www.metaboanalyst.ca) [51]. The following analyses were performed by R software (http://www.r-project.org, version 3.5.3). The differences between each time point of the normal diet group and the corresponding HFD group and among four subgroups of the normal diet group and the HFD group were determined by one-way or two-way ANOVA followed by Fisher’s LSD multiple comparison test and corrected by the Benjamini and Hochberg method to control the false discovery rate (FDR). Fold change (FC) was calculated by comparing the highest and lowest concentrations at each time point in the normal diet group and the HFD group. The ‘corrplot’ package was used to calculate correlation coefficients between samples at different time points. Canonical correlation analysis was conducted between the expression of circadian genes and concentration of metabolites involved in purine catabolism.

### 4.6. qRT-PCR Analysis

Total RNA in the liver was isolated using RNAiso Plus (Takara Bio Inc, Dalian, China), and mRNA was quantified by measuring the absorbance at 260 nm and diluted to 0.5 μg/μL. Then the mRNA was reverse transcribed with RT-PCR kits (Takara Bio Inc., Otsu, Japan) using a CFX96 real-time RT-PCR detection system equipped with a C1000 thermal cycler (Bio-Rad, Hercules, CA, USA). Gene expression levels were determined by SYBR green-based real-time-PCR, and the mRNA levels of GAPDH were used for internal normalization of each sample. The primer sequences used for qRT-PCR are listed in Table 2.

## 5. Conclusions

The high-throughput technique of metabolomics has been expanded to a myriad of biochemical and medical studies. In this study, we sought to discover the most influenced metabolites related to circadian oscillations in response to the high fat diet-induced obesity via metabolomics, and found that metabolites involved in carbohydrate metabolism, fatty acid metabolism, amino acid metabolism and purine catabolism were under circadian control and that their rhythmic cycling can be modulated by an abnormal metabolic status, namely, obesity. We then selected the most oscillated pathway, purine catabolism, and showed that the expression of *Xor* and *Uox*, the key enzyme involved in purine catabolism, can explain the corresponding metabolic changes. The oscillation of *Xor* was further discovered to be strongly correlated with circadian clock gene expression, indicating intriguing relationships between metabolism and circadian control that await future exploration. Moreover, timing drug administration with the personal nutritional status and circadian clock of patients may improve drug effectiveness.

## Figures and Tables

**Table 1:** Nutritional composition of AIN-93M and the high-fat diet.

	AIN-93M	High-Fat Diet
	kcal/%	kcal/%
Protein	15	16
Carbohydrate	76	25
Fat	9	59
Total	100	100
Kcal/gm		
	kcal	kcal
Casein	560	793
Corn starch	1983	0
Maltodextrin	500	880
Sucrose	400	363
Soybean oil	360	306
Lard	0	2757
Cellulose	0	0
Mineral mix	0	0
Vitamin mix	40	57
L-cystine	7	0
Choline bitartrate	0	0
*t*-Butylhydroquinone	0	0
Cholesterol	0	0
Total	3850	5156

**Table 2:** Primers used in qRT-PCR analysis.

Primer	Forward Primer Sequence (5′-3′)	Reverse Primer Sequence (5′-3′)
*GAPDH*	TGACGTGCCGCCTGGAGAAA	AGTGTAGCCCAAGATGCCCTTCAG
*Xor*	AAAGGACCAGACGATTGCTCC	TCACACGTTCCCCTTCAAAAC
*Uox*	GGCCCTATGACAAAGGTGAA	GCAGCAAAACCTCTTCCTTG
*Bmal* *1*	GGACTTCGCCTCTACCTGTTCA	AACCATGTGCGAGTGCAGGCGC
*Clock*	CACTCTCACAGCCCCACTGTAC	CCCCACAAGCTACAGGAGCAGT
*Rev-erbα*	CTACTGGCTCCCTCACCCAGGA	GACACTCGGCTGCTGTCTTCCA
*Cry1*	AGCGCAGGTGTCGGTTATGAGC	ATAGACGCAGCGGATGGTGTCG
*Cry2*	AAGAAGCCCGCGGTGGCTGTGA	CCGTTCCAAGTGCTTGTCCAGG
*Per1*	AACGGGATGTGTTTCGGGGTGC	AGGACCTCCTCTGATTCGGCAG
*Per2*	TGATCGAGACGCCTGTGCTCGT	CTCCACGGGTTGATGAAGCTGG

## Supplementary Materials

The following are available online. Figure S1. High-fat diet induced obesity, hyperglycaemia and hyperlipemia in mice. Figure S2. Venn plot showed the oscillated compounds overlapping between the normal diet group (C) and the HFD group (H). Figure S3. The fitted curves of metabolites involved in purine catabolism. Figure S4. The relative amount of metabolites in the serum of mice from the normal diet group (C) and the high-fat diet group (H) at ZT2, ZT8, ZT14 and ZT20, respectively. Data are represented as the mean ± SD. (A)–(C) show the relative abundance of identified metabolites involved in glucose metabolism, fatty acid metabolism and amino acid metabolism within a 24-h cycle, respectively. *p* value in blue colour, one-way ANOVA of four groups under the normal diet; *p* value in red colour, one-way ANOVA of four groups under the HFD diet. Figure S5. The fitted curves of metabolic enzymes and clock genes.
