# Supplementary figures and images for: Purine Catabolism Shows a Dampened Circadian Rhythmicity in a High-fat Diet-Induced Mouse Model of Obesity
Source: Molecules. 2019 Dec 10;24(24):4524. doi: 10.3390/molecules24244524 (PMC6943701; doi:10.3390/molecules24244524)

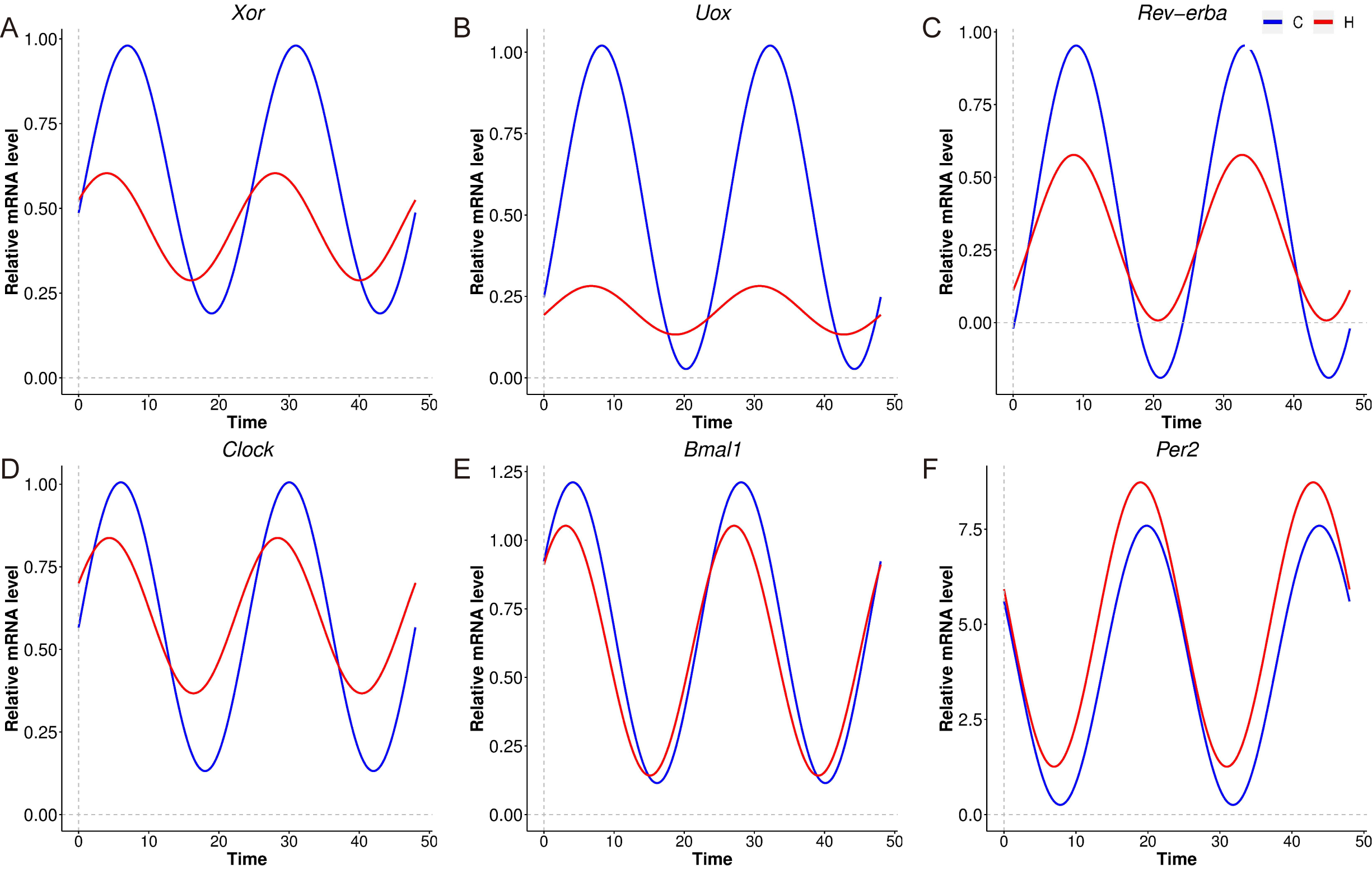

Supplement: Supplementary file 1 [file molecules-24-04524-s001.zip › Supplementary Figure S5.tif]

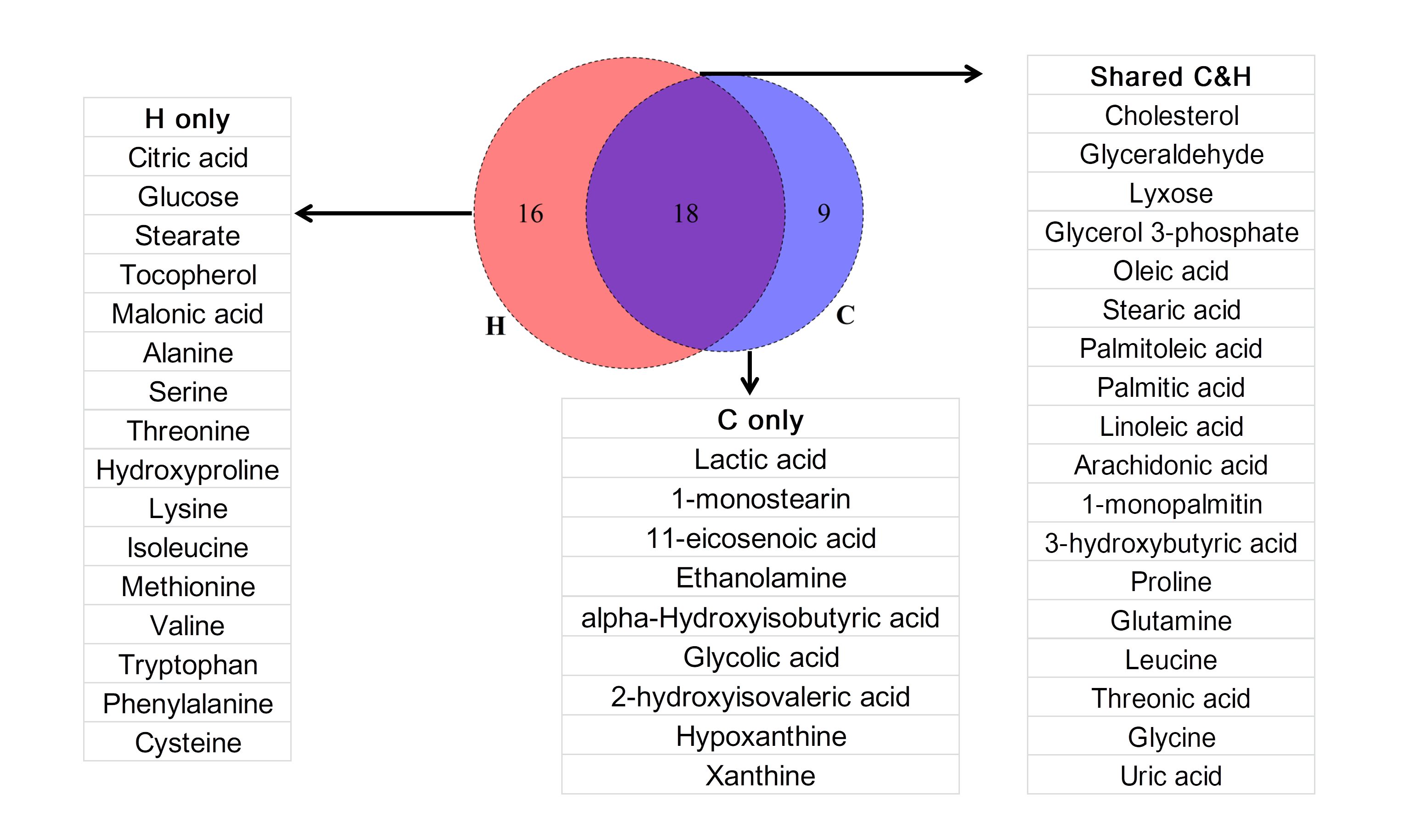

Supplement: Supplementary file 1 [file molecules-24-04524-s001.zip › Supplementary Figure S2.tif]

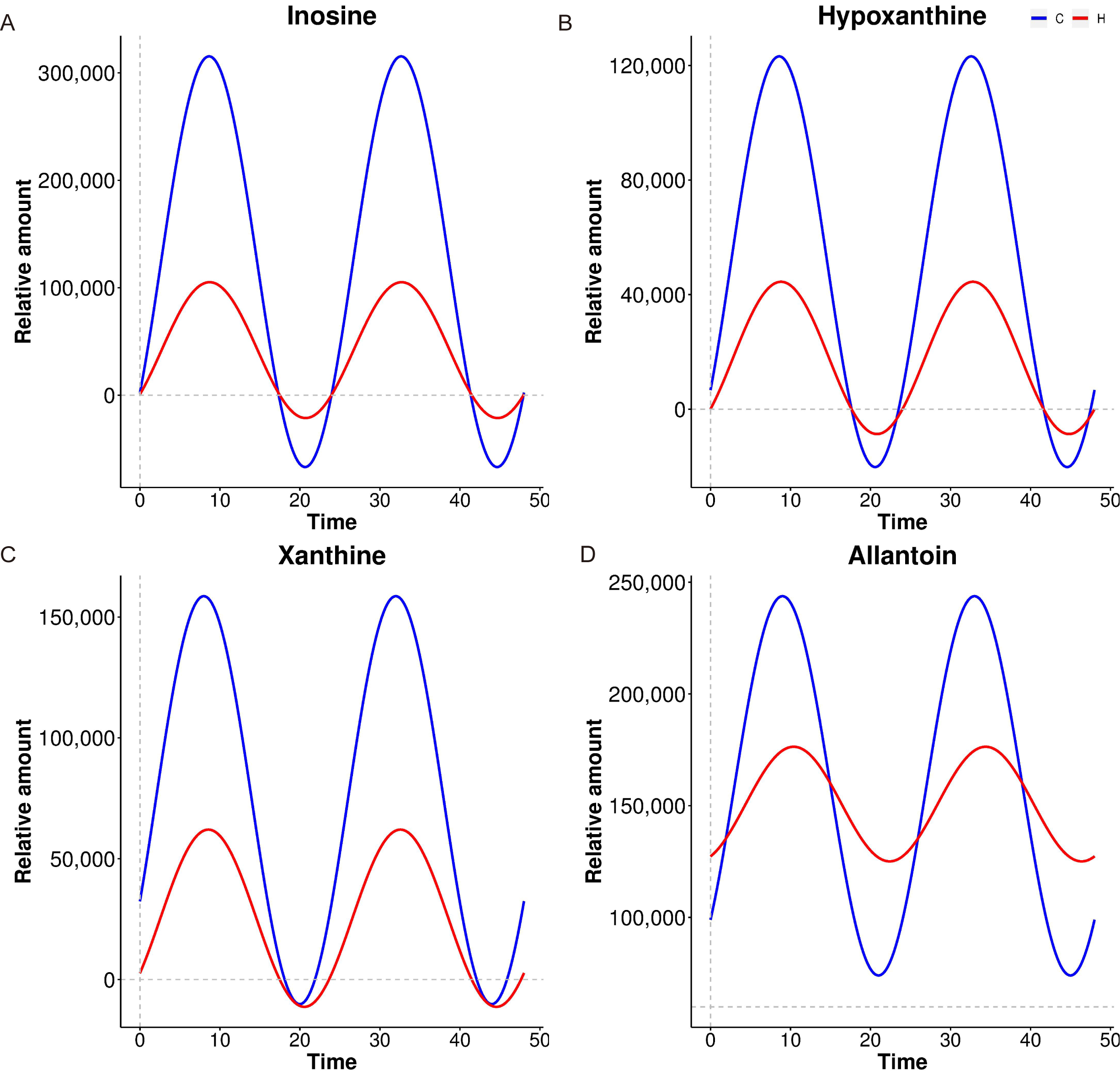

Supplement: Supplementary file 1 [file molecules-24-04524-s001.zip › Supplementary Figure S3.tif]

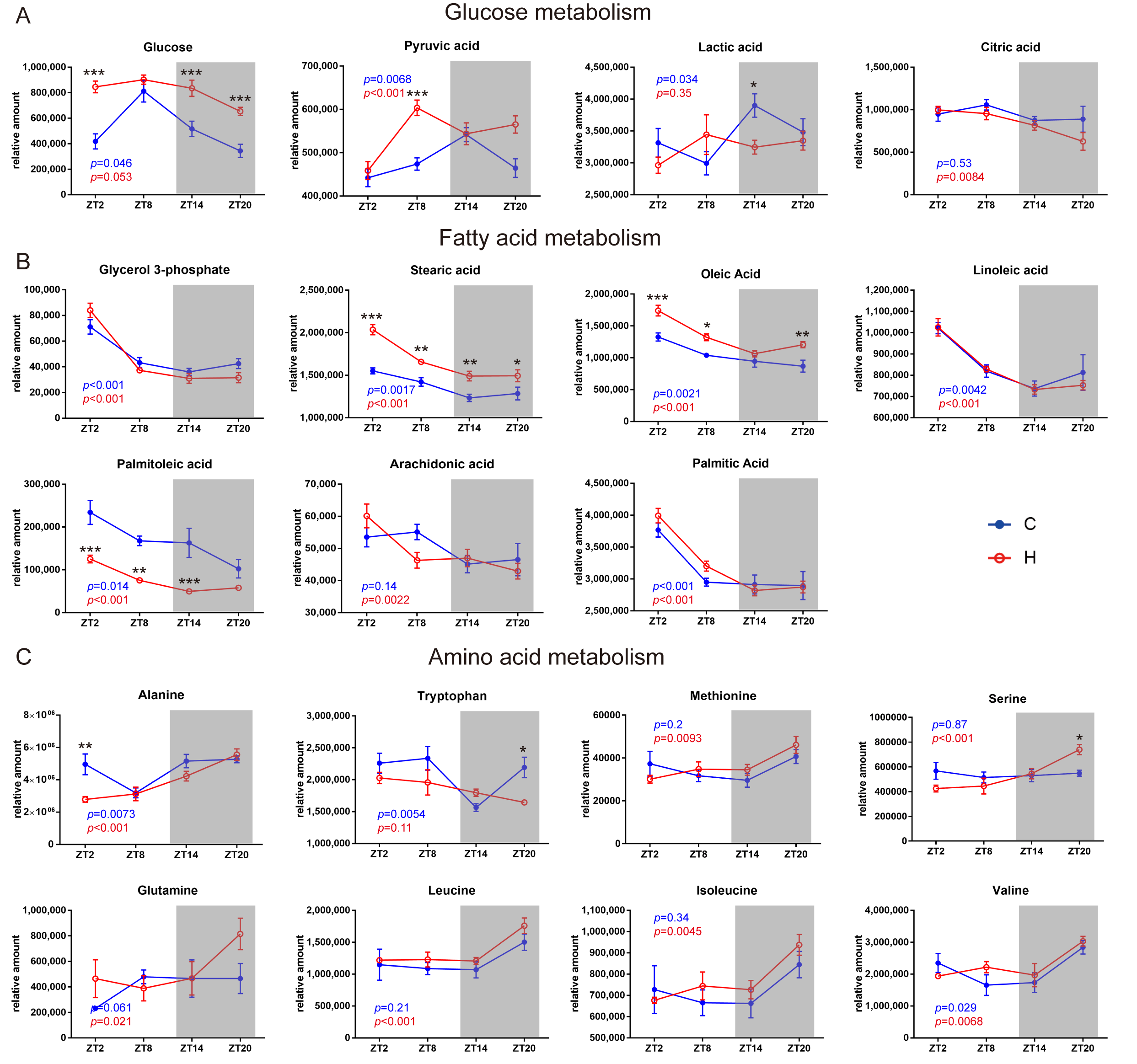

Supplement: Supplementary file 1 [file molecules-24-04524-s001.zip › Supplementary Figure S4.tif]
